# What constitutes a neglected tropical disease?

**DOI:** 10.1371/journal.pntd.0008001

**Published:** 2020-01-30

**Authors:** Peter J. Hotez, Serap Aksoy, Paul J. Brindley, Shaden Kamhawi

**Affiliations:** 1 Departments of Pediatrics and Molecular Virology & Microbiology, Texas Children’s Hospital Center for Vaccine Development, National School of Tropical Medicine, Baylor College of Medicine, Houston, TX, United States of America; 2 Hagler Institute for Advanced Study at Texas A&M University, College Station, TX, United States of America; 3 Department of Biology, Baylor University, Waco, TX, United States of America; 4 James A Baker III Institute of Public Policy, Rice University, Houston, TX, United States of America; 5 Scowcroft Institute of International Affairs, Bush School of Government and Public Service, Texas A&M University, College Station, TX, United States of America; 6 Department of Epidemiology of Microbial Diseases, Yale School of Public Health, New Haven, CT, United States of America; 7 Department of Microbiology, Immunology, and Tropical Medicine, George Washington University School of Medicine, Washington, DC, United States of America; 8 Vector Molecular Biology Section, Laboratory of Malaria and Vector Research, National Institute of Allergy and Infectious Diseases, National Institutes of Health, Bethesda, MD, United States of America; Hospital Universitário Professor Edgard Santos, BRAZIL

## Abstract

The World Health Organization (WHO) currently classifies 20 diseases and conditions as neglected tropical diseases (NTDs). However, since its inception in 2007, *PLOS Neglected Tropical Diseases* has considered an expanded list that includes additional diseases with the chronic and/or debilitating, and poverty-promoting features of NTDs. Described here is an update of our current scope, which attempts to embrace all of the NTDs, and a discussion of the status of some of the more debated medical conditions in terms of whether or not they constitute an NTD.

## Introduction: The birth of the NTD ‘brand’

The framework of NTDs began in the early 2000s after a group of “other diseases”, highlighted within Millennium Development Goal 6, was rebranded based on a set of common features. The original NTDs were all chronic and debilitating infections, most of which were also parasitic diseases, with the added component that they mostly occurred among the extreme poor. In 2005, an initial list of 13 conditions was published in *PLOS Medicine*, two years before *PLOS Neglected Tropical Diseases* began, focused exclusively on the major NTDs of sub-Saharan Africa (Box 1) [1].

Box 1. Original list of 13 African Neglected Tropical Diseases and Their Major Etiologic Agents published in PLOS Medicine in 2005*

**Protozoan Infections**

African trypanosomiasis                    *Trypanosoma gambiense*

                                                                     and *T*. *rhodesiense*

Visceral leishmaniasis (Kala-azar)                *Leishmania donovani*

**Helminth Infections**

Soil-transmitted helminth infections

Ascariasis                                                           *Ascaris lumbricoides*

Trichuriasis                                                      *Trichuris trichiura*

Hookworm infection               *Necator americanus*

Schistosomiasis

Urinary schistosomiasis                                            *Schistosoma haematobium*

Hepatobiliary schistosomiasis             *Schistosoma mansoni*

Lymphatic filariasis                                                 *Wuchereria bancrofti*

Onchocerciasis                                                 *Onchocerca volvulus*

Dracunculiasis                                                 *Dracunculus medinensis*

**Bacterial Infections**

Trachoma                                                                     *Chlamydia trachomitis*

Leprosy                                                                      *Mycobacterium leprae*

Buruli ulcer                                                           *Mycobacterium ulcerans*

* Adapted from [1]

Later, the WHO expanded the 2005 list to include conditions they considered to be of global public health importance (Table 1). Among the helminth infections, they added echinococcosis, foodborne trematodiases, and taeniasis/cysticercosis. Chagas disease was added to the list of protozoan infections, while yaws (endemic treponematoses) was added to bacterial infections. A group of fungal deep mycoses was also added. Viral infections were not on the original list, but the WHO added two arbovirus infections–dengue and chikungunya, as well as rabies. They also added scabies and other ectoparasites, in addition to snakebite envenomation [2].

**Table 1:** Listing the world’s NTDs by WHO and PLOS Neglected Tropical Diseases. NTDs on the cusp refer to conditions that might be added downstream.

Classes of NTDs	NTDs recognized by WHO [2]	NTDs recognized by *PLOS Neglected Tropical Diseases* [3]	NTDs on the cusp
**Helminth infections (and their vectors)**	- Dracunculiasis - Echinoccocosis - Foodborne Trematodiases - Lymphatic filariasis - Onchocerciasis - Schistosomiasis - Soil-transmitted helminthiases (Ascariasis, Hookworm Diseases, Trichuriasis, Strongyloidiasis) - Taeniaisis/Cysticercosis	All human helminth infections including but not restricted to: - Dracunculiasis - Echinococcosis - Foodborne Trematodiases - Loiasis - Lymphatic Filariasis - Onchocerciasis - Other food-borne helminthiases, including Trichinosis, Anisakiasis, Gnathostomiasis, etc. - Schistosomiasis - Soil-transmitted helminthiases (Ascariasis, Hookworm Diseases, Trichuriasis, Strongyloidiasis) - Taeniasis-Cysticercosis - Toxocariasis and other larva migrans, e.g. *Baylisascaris*	- None (all human helminth infections are currently considered)
**Protozoan infections** **(and their vectors)**	- Chagas disease - Human African trypanosomiasis - Leishmaniasis	- Amebiasis including *Naegleria* - Babesiosis - Balantidiasis - Chagas Disease - Giardiasis - Human African Trypanosomiasis - Leishmaniasis - *Plasmodium vivax* and other non-*P*. *falciparum* malarias	- Blastocystiasis* - Cryptosporidiosis and cyclosporiasis - Toxoplasmosis
**Bacterial infections (and their vectors)**	- Buruli ulcer - Leprosy (Hansen’s disease) - Trachoma - Yaws (Endemic treponematoses)	- Bartonella - Bovine Tuberculosis in Humans - Buruli Ulcer - Cholera - Enteric pathogens (Shigella, Salmonella, *E*. *coli*) - Leprosy - Leptospirosis - Melioidosis - Relapsing Fever - Trachoma - Yaws and other tropical treponematoses (Bejel, Pinta) - Q fever	- Group A Streptococcal disease
**Fungal infections**	- Mycetoma, chromoblastomycosis, and other deep mycoses	- Mycetoma, chromoblastomycosis, and other deep mycoses - Paracoccidiomycosis	- Cryptococcosis - Histoplasmosis
**Viral infections (and their vectors)**	-Dengue and Chikungunya -Rabies	- Arboviral infections including Dengue, Chikungunya, Zika, Japanese encephalitis, Jungle yellow fever and others - Enterovirus 71 and related viruses - HTLV-1, HTLV-2 and other non-HIV retrovirus infections - Rabies - Rift Valley fever - Viral hemorrhagic fevers	- *Henipavirus* species e.g. Nipah virus
**Ectoparasitic infestations**	- Scabies and other ectoparasites	- Scabies, Myiasis, and other ectoparasites	- None
**Non-infectious diseases or conditions**	- Snakebite envenoming	- Podoconiosis - Snakebite envenoming	- Sickle-cell Anemia

* The taxonomic position of the stramenopile-like, unicellular protist *Blastocystis* remains uncertain.

### An expanded NTD list for *PLOS Neglected Tropical Diseases*

At *PLOS Neglected Tropical Diseases*, the editors highly support the WHO list but also recognize that the community of NTD investigators conduct research and public health efforts on an expanded group of conditions that still qualify as NTDs due to evidence that they constitute chronic and debilitating conditions disproportionately affecting populations living in extreme poverty [3]. Table 1 lists the NTDs currently recognized by *PLOS Neglected Tropical Diseases* compared to those recognized by the WHO.

Among the helminth infections, *PLOS Neglected Tropical Diseases* largely considers just about any helminthic disease of humans. In some cases, we even consider important veterinary helminthiases if they are linked to human zoonotic disease. In our scope, we specifically mention some important conditions that are not included in the WHO list, including loiasis, strongyloidiasis, and toxocariasis (and other larva migrans syndromes), which represent prevalent, high disease burden, human illnesses.

For protozoan infections, we include some key intestinal infections including amebiasis, babesiosis, giardiasis, and others. *PLOS Neglected Tropical Diseases*, along with the WHO and some other international agencies, does not consider *Plasmodium falciparum* malaria to be an NTD. Nevertheless, *PLOS Neglected Tropical Diseases* recognizes that *P*. *vivax* and other non-*P*. *falciparum* malarias—classically non-fatal but debilitating tropical infections–are understudied relative to falciparum malaria. Therefore, we consider and publish papers on non-falciparum malaria.

For bacterial diseases, the *PLOS Neglected Tropical Diseases* list is notable for adding cholera and other enteric diarrheal pathogens not considered as NTDs by the WHO, in addition to bartonellosis, bovine tuberculosis, leptospirosis, melioidosis, relapsing fever and Q fever, each an important disease in resource-poor regions. While we consider yaws and other tropical treponematoses as NTDs, we are not ordinarily reviewing or publishing syphilis papers unless there are unusual or specific circumstances to justify this.

For viral infections, we consider just about all arbovirus infections if they affect low- and middle-income countries, in addition to Ebola and other viral hemorrhagic fevers, as well as rabies. We now also consider enterovirus 71 or some of the enterovirus infections, as well as HTLV-1 and HTLV-2 infections, if they specially relate to resource-poor countries. Both the WHO and *PLOS Neglected Tropical* Diseases lists include ectoparasitic infestations, especially scabies. We also consider two non-infectious conditions–podoconiosis and snakebite envenoming.

Beyond these NTDs, *PLOS Neglected Tropical Diseases* also considers the important nutritional links underlying NTDs, and co-infections between NTDs and HIV/AIDS, malaria, and tuberculosis. We are also enthusiastic about publishing on the social sciences or public policy if these aspects pertain to neglected diseases.

### Other infections “NTDs on the cusp”

We also wish to highlight here some of our more thought-provoking, interesting, and polemical ongoing discussions regarding specific conditions and diseases, especially some that are most debated in terms of whether they truly represent NTDs. Factoring into deliberations and decisions on whether to include a specific disease or condition within the scope of *PLOS Neglected Tropical Diseases* is the availability of disease burden estimates for that specific condition, and if that burden occurs in resource-poor settings. Delays in accepting submissions in some topics may reflect a lack of editorial expertise to appropriately review and handle such papers, but we will strive to acquire that expertise in order to serve the needs of our community.

Regarding protozoan infections, emerging evidence from the Global Enteric Multicenter Study (GEMS) clearly implicates cryptosporidiosis as an important NTD, especially of young children. Therefore, *PLOS Neglected Tropical Diseases* will add this disease to our list [4]. However, the journal is less interested in cryptosporidiosis outbreaks in North America and Europe due to direct water contamination unrelated to poverty. Similarly, toxoplasmosis is traditionally considered a disease of North America and Europe, but an evidence base is building for high rates of disease transmission in Africa and Latin America. For that reason, toxoplasmosis as it pertains to low-resource countries would be considered relevant. Blastocystiasis is a disease caused by an organism with an unusual phylogeny, and there is controversy whether it constitutes an actual pathogen. However, we have an interest in these discussions and are willing to consider papers on this topic.

For bacterial infections, we currently consider those diseases that disproportionately affect impoverished populations. Relative to the WHO list, adding relapsing fever, leptospirosis, bartonellosis, cholera, and melioidosis to the *PLOS Neglected Tropical Diseases* list was a relatively straightforward decision because of their global public health impact. However, adding *Salmonella*, *Shigella*, and other enteric bacterial infections occurred with the understanding that we would focus on topical papers relevant to the disease-endemic countries of poverty. We now receive occasional correspondence regarding group A streptococcus and its importance in the pathogenesis of rheumatic fever, glomerulonephritis, and other conditions of the tropics. Given that scabies is a major predisposing factor to streptococcal infections and disease sequelae, increasingly we are willing to consider papers on this topic. Deep fungal infections are now a prominent component of the *PLOS Neglected Tropical Diseases* papers. We are also willing to consider papers on cryptococcosis and histoplasmosis if they specifically pertain to disease in the setting of extreme poverty.

For viral infections, the scope of *PLOS Neglected Tropical Diseases* is already larger relative to the WHO list, especially for arboviral infections. We agree that some zoonotic viruses, such as henipaviruses that include Nipah and Hendra viruses, represent emerging tropical disease pathogens, and we are working to expand our editorial expertise to handle and review these papers. At present, we are willing to consider papers on these topics on a case-by-case basis.

Finally, we recognize that sickle cell anemia in many respects resembles an NTD in its clinical and epidemiologic features. We have published a thoughtful editorial making the case why sickle cell anemia might be considered as an NTD [5]. At this time, however, there are already several eminent hematology journals better equipped to review papers on this topic.

Ultimately, the editors at *PLOS Neglected Tropical Diseases* are open to new ideas and articles about diseases not on the current list. Please note that NTDs are not rare diseases, and therefore rare diseases are beyond the scope of this journal.

### Final comment

*PLOS Neglected Tropical Diseases* is a community journal, and in that context, it is important for the editors to be responsive to the NTD scientific community regarding our current and future scope. We very much look forward to hearing from you about our current topics and expertise, and whether there are new directions we should consider. As such, we encourage prospective authors to send us a viewpoint or propose an editorial article to make a case that an additional disease, illness, or condition qualifies as an NTD. These articles are generally well received, are impactful and can serve to drive the field forward. In this spirit, we would also like to emphasize that *PLOS Neglected Tropical Diseases* focuses on human disease, not infections that only afflict livestock and other animals unless these diseases also impact human health and represent a public health problem. Further, we would like to reiterate that the focus of our journal is on translational or epidemiological studies, and less so on pure, basic molecular research not associated to improvement of human health.

The scope of *PLOS Neglected Tropical Diseases* will always be dynamic. We plan to revisit the scope of the journal every five years or so. Once the editorial board arrives at a consensus that an infection or condition qualifies as an NTD, we will strive to bring onboard new expertise to ensure that all NTDs find a home at *PLOS Neglected Tropical Diseases*.
